# The Design, Implementation, and Acceptability of a Telehealth Comprehensive Recovery Service for People With Complex Psychosis Living in NYC During the COVID-19 Crisis

**DOI:** 10.3389/fpsyt.2020.581149

**Published:** 2020-09-08

**Authors:** David A. Lynch, Alice Medalia, Alice Saperstein

**Affiliations:** ^1^ Department of Psychiatry, Columbia University Vagelos College of Physicians and Surgeons and New York-Presbyterian, New York, NY, United States; ^2^ Department of Psychiatry, Columbia University Vagelos College of Physicians and Surgeons, New York, NY, United States

**Keywords:** COVID-19, telehealth, complex psychosis, comprehensive recovery service, implementation

## Abstract

**Introduction:**

The COVID-19 crisis and subsequent stay-at-home orders have produced unprecedented challenges to the dissemination of recovery oriented behavioral health services (RS) that support the treatment of those with complex psychosis (CP).This population has typically been managed with in-person pharmacotherapy and/or RS, with the goals of relieving symptoms, improving life satisfaction and increasing community engagement. COVID-19 related social distancing measures have required rapid shifts in care management, while easing of telehealth regulations has allowed for flexibility to approach RS differently. It is essential to learn from the RS telemedicine implementation experience, so that RSs can maintain care for this vulnerable and needy population.

**Method:**

This paper describes the successful telehealth conversion of a NYC-based, university affiliated RS that serves adults with severe mental illnesses (SMI; *n* = 64). Results focus on the telehealth acceptance rates of the subset of participants with CP (*n* = 23).

**Results:**

The RS continued providing services including intake, care coordination, group psychotherapies, skills training groups, individual skills coaching, and vocational/educational supports. The telehealth conversion rates of the CP subsample indicated that 90% of CP patients accepted telehealth sessions and maintained their specific treatment plans in the virtual format. Mean comparisons between session attendance and cancellations/no-shows during the six-week period before and after telehealth conversion showed no significant differences in service utilization.

**Discussion:**

RSs play an essential role in the treatment of CP and telehealth may prove to be a viable format of care delivery even after the COVID-19 crisis subsides. The multiple factors in the inner and outer treatment setting that contributed to successful conversion to telehealth will be considered along with the challenges that clinicians and patients encountered.

## Introduction

The COVID-19 crisis has challenged the ability of mental health systems to provide the comprehensive services that people with complex psychosis (CP) require to achieve and sustain a rewarding and productive life in the community. While CP is not specifically a DSM-5 or ICD-10 classification, CP is understood as a constellation of neuropsychological, physiological, developmental and behavioral difficulties that, when co-occurring with a psychotic disorder, have a pronounced impact on recovery and overall community functioning (1). Because people with CP must negotiate severe symptoms that may not respond to first line treatments, and often have co-morbid and pre-existing disorders, they benefit from comprehensive supports to achieve optimal functioning. These comprehensive supports typically involve in-person pharmacotherapy and recovery oriented behavioral health services (RS), many of which are provided in a group format. With the onset of the COVID-19 crisis, many in-person psychiatric services were no longer feasible due to public health mandates to social distance and stay at home; hence, people with CP lost the comprehensive care that has been shown effective for promoting community integration (2, 3). RSs, which typically would offer independent living and social skills training, psychological support and symptom management training, employment, educational and social supports, and access to leisure activities, started to only offer long-term injectable medication and telephone check ins. In order to maintain the skills training and recovery services impacted by COVID-19 public health mandates, a radical shift in treatment dissemination was needed and agencies turned to telehealth.

Telehealth, or synchronous telemedicine, utilizes a live videoconferencing format with a two-way audio-visual link between patient and provider. Rapid improvements in technology and access to high speed internet have increased the feasibility and acceptability of telehealth services for people with schizophrenia-spectrum disorders (4–6). Nevertheless, prior to COVID-19, telehealth was not widely disseminated for treatment of people with psychosis. Instead, it was used primarily for interim individual sessions with patients already affiliated with a specific provider, or when the availability of qualified mental health professionals may be limited. Since the onset of the COVID-19 pandemic there have been more reports of telehealth to address mental health (7–11); however, the focus remains on individual sessions and the use of telehealth for people with CP is still poorly understood.

Group-based interventions that target symptoms, promote pro-mental and physical health behaviors and foster interpersonal engagement are a mainstay of RS based psychosocial treatment for CP (12). While there has been increased interest in the use of digital therapies for this population (6, 13), regulations and HIPAA compliant technology to support group based interventions have lagged. With the rapid onset of COVID-19 in New York City, the crisis required expedited modifications to replace in-person visits with a telehealth alternative. Only since March of 2020, with the deregulation of telehealth services, has the use of the synchronized video platforms (e.g., ZOOM, WebEx) that support HIPAA compliant group psychotherapies been permitted in the USA. Thus, there remains limited empirical evidence examining the acceptance and use of telehealth platforms for group-based RS with people with CP (4, 11).

People with serious mental illnesses (SMI), which includes the CP population, are challenging to engage in in-person treatment because of symptom severity, low level of daily functioning and motivation (14). The addition of pandemic related social distancing requirements compounds the challenges of providing an intervention the CP population finds acceptable. There are feasibility concerns about using telehealth, given the cognitive deficits commonly seen in CP, though in medical populations with cognitive deficits, telehealth has been found feasible and acceptable for providing group based psychoeducational interventions (6, 15). To further the understanding of telehealth acceptance in people with CP, the current study examines the service utilization of a CP cohort attending a largely group-based RS prior to and following conversion to telehealth formats.

## Methods

### Description of the Recovery Service Program

This study took place in a private university-affiliated outpatient psychiatric treatment center (www.lieberclinic.com) that provides comprehensive psychosocial and rehabilitation services to adults over the age of 18. The RS uses a recovery-oriented model to offer support for patients with SMI whose primary diagnoses typically include schizophrenia-spectrum disorder, high functioning autism spectrum disorder, and mood disorders. Using shared decision making, each RS participant works with their care coordinator to craft an individualized plan of therapeutic services that address their recovery goals. The RS provides a wide range of evidence-based services which include intake assessment, care coordination, group psychotherapies, skills training groups, individual skills coaching, vocational/educational supports, family services and recreational activities. Treatment is primarily delivered in groups; group interventions include cognitive behavioral therapies (CBT) for specific symptom clusters (e.g., psychosis, anxiety, depression, sleep), dialectical behavior therapy (DBT), Wellness Recovery Action Planning (WRAP), acceptance and commitment therapy (ACT), cognitive remediation (CR), executive functioning skills training, harm reduction, social skills and cognition training and family support. Prior to the COVID-19 pandemic, all clinical services were offered in-person at the clinic, with the exception of the individual skills coaching which was also offered in community settings.

### Process of Telehealth Conversion

Due to local governmental stay at home mandates, the RS underwent a rapid telehealth conversion between March 16, 2020 and March 19, 2020. Following the conversion, no patients were seen in-person. To facilitate the conversion to telehealth, care coordinators communicated with patients and stakeholders that all scheduled sessions would be offered as previously scheduled, but in a synchronous video format. In order to participate, patients completed an additional written consent for telehealth, noting the risks and benefits. All participants were encouraged to maintain their in-person treatment plan (i.e., groups, skills coaching, individual psychotherapy, etc.) via the telehealth platform.

Modified institutional workflows, clinical procedures and technological support promoted a seamless continuity of care (11). The most challenging service element to implement was telehealth group treatment, which was unprecedented given prior regulatory constraints. Clinicians had to learn to manage group process and content virtually, and each participant needed access to and basic education in the use of telehealth platforms (e.g., ZOOM, WebEx). Content of group sessions was revised to promote engagement using telehealth and include opportunities to address COVID-related concerns. See **Table 1** for the preparatory activities associated with successful telehealth conversion.

**Table 1:** Preparatory work for telehealth conversion and methodology of implementation.

Workflows	Technology	Stakeholders	Considerations
Workforce regulations Notification of Telehealth transition	Email, WebEx, phone calls Email, phone call, text message	Clinicians, clerical staff, administrators Patient, family, care coordinators, external treatment providers	Consider factors to support and capture work from home productivity Utilize communication methods with highest likelihood of visibility; document attempts at communication
Consents/Telehealth Terms & Conditions	EMR, email	Patient, family	Signed/consented *via* email prior to telehealth use; uploaded/documented into EMR
Telehealth technology orientation (staff)	WebEx, Zoom	Administration, Clinical staff Information Technology (IT)	Provide virtual trainings of the features and functionality of telehealth platforms
Telehealth technology orientation (patient)	WebEx, Zoom, telephone, iPad		Provide as needed individualized instruction about telehealth platforms
Scheduling a group/individual session	EMR, Zoom, WebEx	Clinic administration, clinical staff	When possible, maintain the schedule and timing of services; maintain strong administrative support
Group expectations, i.e., “web-iquette”	Zoom, WebEx	Patients, clinical staff	Determine group rules and expectations that promote safety and confidentiality; proactively address interruptive behaviors (e.g., muting mic when not speaking, closing apps/programs that may be distracting, etc.)
Adapting group content	Zoom, WebEx	Clinical Staff	Familiarize yourself with the screensharing, annotation and document sharing functionality built-in to telehealth platform
Crisis Management	EMR, Zoom, WebEx	Clinical staff, on-call clinician	Utilize digital formats of safety planning; consider reviewing telehealth specific risk assessment practices
Billing	EMR	Clinic administration, clinical staff	CPT codes with a synchronous telehealth modifier

EMR, electronic medical record.

### Sample

This study focuses on data from a subsample of CP patients within the overall SMI sample enrolled at the RS (*n* = 64) six weeks post telehealth conversion. The cohort included CP (*n* = 23) and non-CP patients (*n* = 41) who, due to the COVID-19 crisis, were required to choose whether to continue their treatment in a telehealth format. Patients met inclusion for the CP subsample by having a documented psychotic disorder and at least one of the following: past or concurrent substance use, pre-morbid developmental disorders (e.g., Autism Spectrum Disorder), concurrent physical health conditions and past or concurrent mood disorder symptoms. The identification of psychotic episodes was based on DSM-5 criteria, which include the presence of one or more of the following: delusions, perceptual disturbances, disorganized speech, abnormal behavior, and negative symptoms. Diagnoses were established by psychologists and psychiatrists at intake and reported in the electronic medical record (EMR).

### Method of Data Collection and Analysis

As part of a program evaluation initiative, telehealth acceptance, intakes, session attendance, diagnoses, age and race were determined using a comprehensive chart review of the EMR for all RS participants. Data was extracted by DL and verified by AM, who both serve as clinicians in the RS, with access to the EMR. A de-identified database was created for program evaluation to determine trends in service utilization, overall attendance and missed sessions from the six weeks prior to the telehealth conversion to the six weeks following the conversion. Identifiable private information and all possible linkages to identifiable information were removed in the database used for this research. The governing Institutional Review Board determined that criteria for human subjects research, under 45 CFR 46, were not met and exempted this study from further review.

All analyses were conducted using native R packages. Within-subject and between-subject mean comparisons were conducted using Welch’s *t*-tests when comparison groups had unequal variances. Chi-square analyses were used to compare frequencies between groups, using the Yates’ continuity correction to account for small values.

## Results

### Sample Characteristics

The CP subsample composition was primarily composed of white/Caucasian (88%) males (74%), with an average age of 32.6 (*SD* = 12) years. They differed from the non-CP individuals in the overall sample by virtue of having a psychotic disorder and being significantly older. See **Table 2** for non-CP and CP subsample demographics.

**Table 2:** Demographics and service utilization of CP subsample versus non-CP cohort (*N* = 64).

	CP Cohort	Non-CP Cohort	Test statistic (*df*)	*p*
	(*n* = 23)	(*n* = 41)		
Age				
Mean (*SD*)	32.6 (12)	26.1 (9.49)	*t* = -2.37 (35.9)	*
				
Gender *N* (%)				
Male	17 (74)	20 (49)	*X^2^* = .61 (1)	
Female	5 (22)	17 (41)		
Non-Binary	1 (4)	4 (10)		
				
Race/ethnicity *N* (%)				
White/Caucasian	20 (88)	39 (95)	*X* ^2^ = .01 (1)	
Black/African American	1 (4)	0		
Hispanic, Latinx	1 (4)	1 (2.5)		
Asian	1 (4)	1 (2.5)		
				
Telehealth acceptance				
*n* (%)	18 (90)	39 (95)	*X^2^* = 0.01 (1)	
				
Sessions Attended - Pre-conversion				
mean (*SD*)	18.6 (14.54)	24.57 (13.58)	*t* = 1.51 (36.8)	
				
Sessions attended—Post-conversion				
mean (*SD*)	21.33 (13.48)	23.6 (17.53)	*t* = 0.56 (50.8)	
				
Sessions Missed — Pre-conversion				
mean (*SD*)	3.85 (4.44)	6.92 (6.16)	*t* = 1.73 (38.5)	
				
Sessions missed — Post-conversion				
mean (*SD*)	2.9 (3.16)	4.26 (3.84)	*t* = 1.46 (48.4)	
				
				

*p < 0.05. Welch two sample t-test used for comparisons with unequal variances. Chi-square test used with Yates’ continuity correction.

### Telehealth Implementation

The RS continued providing all services except community-based coaching via telehealth. A coordinated effort between administration and clinical staff allowed for a nimble response to rapidly evolving regulatory changes, so that new workflows could be created (11). See **Table 1** for implementation considerations. Although all participants at the RS had access to technology, the individuals in the CP cohort were most likely to require extensive individualized training via telephone in order to use the technology, in some cases requiring the support of virtual skills coaching. Some clients struggled with virtual etiquette (e.g., appropriate camera background, dress, comportment); they were asked to help staff draft a guide to “Web-iquette,” which was periodically reviewed at the start of groups or discussed on an individual basis. Maintaining attention in the virtual session was a common problem which was addressed in three ways: 1. clinicians problem-solved with clients to minimize on-screen distractions (e.g., web browser, mobile notifications, etc.); 2. clinicians used screen-sharing features and interactive activities (e.g., mindfulness exercises, ice breakers, etc.) that provided additional opportunity for engagement; 3. for groups lasting longer than one-hour, brief breaks and “check-ins” were provided.

### Acceptance and Utilization Data

**Figure 1:**
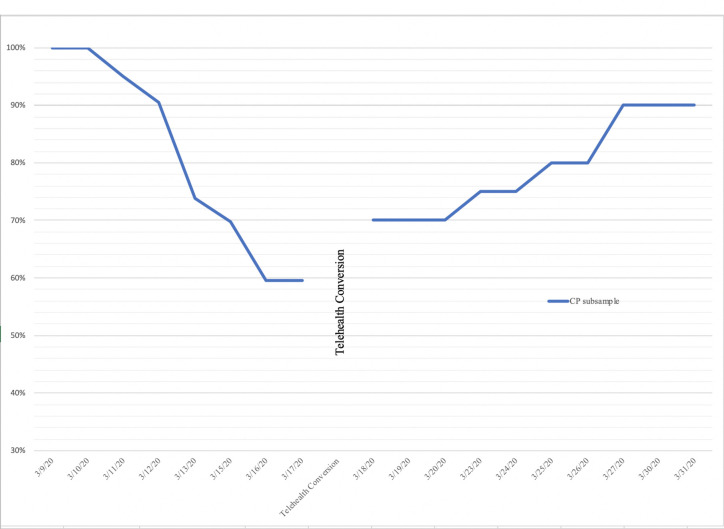
Percentage of actively enrolled CP participants prior to and after telehealth conversion (*n* = 23).

During the 12-week study timeframe, the CP subsample was psychiatrically stable; there were no psychiatric decompensations or referrals to a higher level of care. The telehealth acceptance rates of the CP subsample indicated that 90% (*n* = 18) of the *n* = 20 CP patients enrolled at the time of conversion agreed to telehealth sessions within ten days of the service transition, and maintained their specific treatment plans virtually. Two patients (10%) opted out of telehealth services and three patients entered the RS following the telehealth conversion (comprising a total of *n* = 23 CP patients seen during the study timeframe). In the six weeks prior to the telehealth conversion, the CP subsample attended an average of 18.6 (*SD* = 14.54) sessions (3.1/week) while missing an average of 3.85 (*SD* = 4.44) sessions. Following telehealth conversion, those who accepted telehealth (*n* = 21) attended an average of 21.33 (*SD* = 13.48) sessions (3.5/week), while missing an average of 2.9 (*SD* = 3.16) scheduled sessions. Mean comparisons between session attendance and cancellations/no shows during the six-week period before and after telehealth conversion showed no significant differences in service utilization. There were no significant differences between the CP subsample and the non-CP cohort with regards to telehealth acceptance and service utilization. See **Table 2** for service utilization and mean comparisons. See **Figure 1** for telehealth acceptance rates over the study timeframe.

## Discussion

Recovery oriented behavioral health services (RS) play an import role in the treatment of people with complex psychosis (CP), providing comprehensive supports to help participants achieve optimal functioning. While the use of telehealth technology has been explored in the field, there has been limited empirical reporting on rapidly converting an entire RS to the telehealth format (8, 16). Before the COVID-19 pandemic, RSs were offered in-person, with the belief that the socialization afforded by group interactions would facilitate community engagement. COVID-19 related social distancing measures have required a shift in how care is delivered and, for the first time, USA regulations allowed group interventions to be provided as a telehealth service. For services that converted to providing groups via telehealth, many novel questions were raised. Would participants with CP find telehealth RS acceptable and agree to participate? Would they participate at the same rate in telehealth services as in person services? Would people with CP differ from others with severe mental illness (SMI) in their acceptance of telehealth RS? What are the factors in the inner and outer treatment setting that contribute to successful conversion to telehealth? This study used the experience of one inner city RS that adapted to telehealth for people with CP, in order to provide some answers to these questions.

This retrospective study found that the vast majority of participants with CP (90%) agreed to participate in telehealth services within ten days of service transition. The RS also enrolled an additional three CP participants in the six weeks following the telehealth conversion. Comparisons of service utilization of in-person and telehealth sessions showcased that the rates of attendance and missed appointment did not change. Following the telehealth conversion, participants and clinicians sought to maintain individualized treatment plans and group schedules whenever possible, which may have contributed to the high acceptance rates and unchanged service utilization. CP participant service utilization and telehealth acceptance did not differ compared to the rest of the SMI cohort within the RS. When considered together, the study suggests that telehealth services are both feasible and readily acceptable for participants with CP.

Successful telehealth transition provided essential services for a CP subsample receiving specialized services and frequent clinical contact. Outer setting factors that facilitated this RS telehealth conversion included relaxation of pre COVID-19 government regulations that prohibited interstate telehealth services and the use of the platforms that enabled group therapy. Some of the CP participants left the state in order to shelter in place with families or in less populated areas, and with deregulation of interstate telehealth they were able to continue care at the RS. This virtual care was in turn facilitated by government deregulation of telehealth platforms needed to run groups. The RS relied on platforms like ZOOM and WebEx that adapted to provide HIPAA-compliant access.

Acceptance of telehealth conversion is an active area of study, as it informs service delivery considerations both during emergency situations and in stable conditions. The acceptance rate (90%) in this RS is much higher than a previous report that 52% of SMI patients transitioned to telehealth services (17). Inner setting factors that may have facilitated acceptance of the telehealth conversion in this RS range from administrative organization to clinician practice and participant characteristics. In terms of administrative factors, the overarching faculty practice and RS-specific administrations were nimble in responding to a rapidly changing regulatory and public health landscape. Pre-existing strong communication channels connecting clinicians to administration facilitated rapid telehealth conversion and supported ongoing adaptations to maintain quality care. In terms of clinician practice, individualized support, as needed, emphasized expectations for group membership, mollified anxieties or suspiciousness and resolved technological issues. Group leaders adapted session materials and structure to enhance engagement and address the attention deficits associated with CP. Finally, participant characteristics likely also facilitated the success of the telehealth conversion. As a private largely self-pay clinic, the RS serves participants with higher socioeconomic status (SES). In turn, high SES is associated with increased access to up-to-date technological devices, high speed internet connection and a living space that provides adequate privacy. Together, these inner settings factors addressed the technologic, clinical and administrative challenges to telehealth that have been previously identified (8).

Providing continuity of care, especially in the context of the COVID-19 pandemic, helped maintain crucial mental health services, promote community socialization, and follow social distancing guidelines for this cohort of people with CP. Unlike many studies examining telehealth and in-person sessions, the current study limits selection bias since all enrolled participants were offered telehealth services following the COVID-19 stay-at-home order guidelines. Another strength of this study is the reliance on utilization data to inform our understanding of telehealth acceptance. Since attitudes and intentions do not necessarily translate into their behaviors (18), exclusive reliance on satisfaction surveys may only inform an aspect of acceptance. That said, the lack of direct assessment of provider and user feedback and small sample size could be seen as limitations. Further, patient homogeneity likely contributed, in part, to the high acceptance rate and maintained service utilization. It will be important to continue to examine acceptability over a longer timeframe in order to understand utilization patterns and possible moderating factors. While tragic circumstances have hastened the use of telehealth as the primary format of recovery-oriented treatment, we may be heralding an essential treatment dissemination strategy that continues once the crisis subsides (19). Hence, efficacy and effectiveness trials are needed to compare relevant treatment outcomes between in-person and telehealth treatment formats.

## Data Availability Statement

The raw data supporting the conclusions of this article will be made available by the authors, without undue reservation.

## Ethics Statement

The governing Institutional Review Board determined that this study did not meet criteria for human subjects research and exempted it from further review. Written informed consent for participation was not required for this study in accordance with the national legislation and the institutional requirements.

## Author Contributions

DL contributed to the submission in the following ways: data extraction and management, data analysis, manuscript writing. AM contributed to the submission in the following ways: concept formulation, study design, manuscript writing. AS contributed to the submission in the following ways: ethics oversight and manuscript writing. All authors contributed to the article and approved the submitted version.

## Conflict of Interest

The authors declare that the research was conducted in the absence of any commercial or financial relationships that could be construed as a potential conflict of interest.
